# The lung microbiota: role in maintaining pulmonary immune homeostasis and its implications in cancer development and therapy

**DOI:** 10.1007/s00018-020-03452-8

**Published:** 2020-01-23

**Authors:** Michele Sommariva, Valentino Le Noci, Francesca Bianchi, Simone Camelliti, Andrea Balsari, Elda Tagliabue, Lucia Sfondrini

**Affiliations:** 1 Dipartimento Di Scienze Biomediche Per La Salute, Università Degli Studi Di Milano, via Mangiagalli 31, 20133 Milano, Italy; 2 Molecular Targeting Unit, Department of Research, Fondazione IRCCS, Istituto Nazionale Dei Tumori, via Amadeo 42, 20133 Milano, Italy

**Keywords:** Commensal microorganisms, Respiratory tract, Immune system, Immunological tolerance, Tumor

## Abstract

Like other body districts, lungs present a complex bacteria community. An emerging function of lung microbiota is to promote and maintain a state of immune tolerance, to prevent uncontrolled and not desirable inflammatory response caused by inhalation of harmless environmental stimuli. This effect is mediated by a continuous dialog between commensal bacteria and immune cells resident in lungs, which express a repertoire of sensors able to detect microorganisms. The same receptors are also involved in the recognition of pathogens and in mounting a proper immune response. Due to its important role in preserving lung homeostasis, the lung microbiota can be also considered a mirror of lung health status. Indeed, several studies indicate that lung bacterial composition drastically changes during the occurrence of pulmonary pathologies, such as lung cancer, and the available data suggest that the modifications of lung microbiota can be part of the etiology of tumors in lungs and can influence their progression and response to therapy. These results provide the scientific rationale to analyze lung microbiota composition as biomarker for lung cancer and to consider lung microbiota a new potential target for therapeutic intervention to reprogram the antitumor immune microenvironment. In the present review, we discussed about the role of lung microbiota in lung physiology and summarized the most relevant data about the relationship between lung microbiota and cancer.

## Introduction

The human body is colonized by bacteria and other microorganisms that maintain a symbiotic relationship with the host, creating an ecological community defined as microbiota. In the last few years, the importance of microbiota in participating in different aspects of human physiology is emerging. Bacteria inhabiting the intestine are reported to play crucial roles in the digestion of different types of nutrients, in the production of vitamins and other metabolites and, most importantly, in regulating the immune homeostasis. However, other organs possess their own micriobiota, such as lungs [1].

The discovery of lung microbiota was delayed by the long-held view that the lungs of healthy individuals were sterile and by technical difficulties in collecting samples for the analysis [2]. The new technologies, based on the high-throughput sequencing of the 16S rRNA gene, significantly revolutionized our knowledge in the field. Indeed, one of the first published studies that utilized those genomic techniques finally proved that lungs harbor a wide range of different microbial species that are important in keeping the lungs in a good health status [3].

In the present review, we summarized the most important data available about the lung microbiota mainly focusing on the connections between lung microbiota and cancer.

## Lung microbiota: development and composition in healthy lungs

Lungs are lined by a thin mucus layer with bacteriostatic properties that represents a low-nutrient environment for microbes. Lung microbiota are constituted by approximately 2.2 × 10^3^ bacterial genomes per cm^2^ and their composition depends on three ecological factors: immigration, elimination, and the effects of local growth conditions on the replication rates of community members. Microbiota modifications can be determined by the perturbations in one or more of these factors. At the steady state, the microbial community balance is the result of a dynamic equilibrium maintained by immigration, through microaspiration, and elimination, via cough, mucociliary clearance, and immune system activity. The type and number of microbial species present in the lungs are not homogeneous along the airways, but are influenced by the anatomical site and by local environmental conditions. Factors that impact on commensal bacteria growth and survival, include those common to all microbial ecological niches present in the human body, such as nutrients availability, temperature, pH, oxygen tension, as well as the abundance and activation state of host immune cells. In healthy lungs, the environment is generally inhospitable for bacterial community development, resulting in relatively low bacterial replication rate. The microbial community is continually renewed and replaced, but the majority of microbes involved in these processes belongs to four phyla: *Bacteroidetes*, *Firmicutes*, *Proteobacteria*, and *Actinobacteria*. In healthy humans, *Prevotella*, *Streptococcus*, *Veilonella*, *Neisseria*, *Haemophilus*, and *Fusobacterium* are the most abundant genera in the lungs [2-4].

However, during the occurrence of a lung pathology, the local growth conditions change dramatically, creating permissive niches for the growth of specific bacterial species that are able to adapt to a specific environmental conditions of the altered respiratory tract. The adaptation to a peculiar lung environment confers to these microbes an advantage that overwhelms the influence of immigration and elimination processes on the respiratory ecosystem [5].

## Lung microbiota and pulmonary immune tolerance

Although, the lung immune cells have to patrol the airways to counteract the spread of pathogens, one of their most important duties is to avoid exaggerated and unwanted inflammatory responses to harmless environmental stimuli. Indeed, the lung microenvironment is characterized by high immune “tolerance” that is primarily maintained by subpopulations of alveolar macrophages (AMs) and dendritic cells (DCs) [6]. These cells exert their immunoregulatory properties by inducing the generation of regulatory T cells (*T*_reg_) [7] and by the release of prostaglandin E2 (PGE_2_), tumor growth factor-beta (TGF-β) and interleukin-10 (IL-10) [6]. Increasing evidences indicate that lung microbiota, acting on resident immune cells, have a key role in promoting immune tolerance in lungs.

### Interaction of lung microbiota with local immune cells

Lung immune cells, especially antigen-presenting cells (APCs, i.e., AMs and DCs), and airway epithelial cells are equipped with sensors, the so-called pattern recognition receptors (PRRs), able to recognize molecules of host and microbial origin. PRRs comprise different types of receptors, such as Toll-like receptors (TLRs), NOD-like receptors (NLRs), C-type lectin receptors (CLRs) and protease-activated receptors (PARs), whose activation, upon engagement by their own specific ligands, induces the expression of immune-related genes encoding for inflammatory cytokines, type I interferons, and antimicrobial peptides. Their activation promotes the initiation of innate and adaptive immune response against pathogens [8, 9].

The fact that PRR ligands are shared by both commensals and pathogens raises the question about the ability of lung immune system to promptly respond to an incoming infection. All innate immune sensors are able to distinguish between *danger signals*, leading to the production of pro-inflammatory mediators, and *safety signals*, molecules released by not-damaged tissues, dietary components and commensal microbiota [10]. The discrimination between signals originating from “bad” or “good” bacteria can be achieved through different ways. These mechanisms have been well investigated in the gut, but it is reasonable to speculate that a similar situation can also occur in the lungs.

Aside from the fact that microbiota can act as a mechanism of defense itself preventing the growth and spread of potential harmful microorganisms and, therefore, avoiding their recognition by the immune system [11], the simplest method to achieve tolerance can be defined as “tolerance by exclusion”, since commensal bacteria are largely excluded from the lining epithelium [12, 13]. Due to the presence of mucus, the microbiota, lacking the ability to completely pass through this protective layer, cannot reach the epithelium and therefore cannot be sensed by epithelial PRRs [13]. On the contrary, pathogenic bacteria that possess the so-called virulence factors can easily breach the mucus layer and disseminate through the epithelium and the underlying tissues causing the initiation of an inflammatory response [13, 14]. Moreover, to avoid the recognition of commensal microbes by PRRs, these receptors are not randomly distributed along the mucosal surface, but their expression is strategically compartmentalized in well-defined areas, usually inaccessible to commensal bacteria [15-18]. The restricted expression of PRRs reinforces the “tolerance by exclusion”. In this scenario, the lack of a cross talk between commensal bacteria and PRRs seems to be a prerequisite to maintain intestinal homeostasis, but more recent data challenge this view. Indeed, a growing body of evidence suggests that, in normal conditions, a persistent PRR activation triggered by microbiota-derived signals is essential to preserve the epithelial barrier integrity [19-21]. Therefore, further investigations are needed to fully understand the role of PRRs in governing mucosal regulatory mechanisms. As already mentioned, PRRs signaling is necessary to promote an adequate innate and, eventually, adaptive immune response against invading pathogens, but, in “steady-state” conditions, this signaling is generally suppressed or mitigated to prevent potentially detrimental chronic inflammation. For this reason, PRRs expressed by epithelial cells and immune cells populating the mucosal interfaces are hyporesponsive to their own cognate ligand stimulation, and PRR hyporesponsiveness is the result of the combination of different factors [22, 23]. First, the epithelium and immune cells at the mucosal interface have been found positive for the expression of proteins involved in dampening PRR signaling pathways, so that these cells can finely tune the response to PRR stimulation depending on the stimulus received [22, 23]. Second, after PRR recognition, PRR receptors establish various forms of cooperation with other innate receptors and the nature of these interactions can influence the specificity for a particular ligand and the downstream signaling pathway promoting different cellular response, another way to discriminate between safety and danger signals. Moreover, the final outcome of PRR receptor signals depends also on the milieu of cytokines and other soluble factors that can shape the immune cell reaction to a specific PRR agonist [10]. For example, the immunoregulatory cytokine IL-10, secreted by immune cells, is able to counteract PRR activation [24].

In addition to mechanisms that the human body has developed to maintain tolerance toward commensals, it is worth highlighting that also the microbiota co-evolved with humans implementing several stratagems to avoid immune recognition. It has been reported that, due to peculiar modifications of their chemical composition, signals derived from commensal microbes are less agonistic to PRR compared to those from pathogens [13, 25, 26]. However, it is very unlikely that a microorganism may be recognized on the basis of only one PRR ligand. It is plausible to hypothesize that the “good or bad” bacteria discrimination is based on a plethora of signals recognized by different combination of PRRs, creating a signature that identifies, in a unique way, a specific bacteria.

With particular regard to lung environment, it is emerging that the cross talk between microbiota-derived PRR ligands and airway resident immune cells may represent an important factor to sustain a certain grade of immunosuppression. For instance, TLR tolerance is achieved after persistent TLRs stimulation, implying that previous exposure to a TLR ligand decreases the inflammatory responses to a second challenge with the same ligand or with another one [27-29]. In healthy lungs, APCs and epithelial cells are continuously exposed to these ligands and it is now well known that repeated or chronic exposure to endotoxins causes macrophage, dendritic cells and epithelial cells to acquire a tolerogenic phenotype [30-33].

Moreover, the contribution of microbial stimuli, through PRR receptor stimulation, in maintaining the immune tolerance in lungs is also testified by the fact that lung DCs primed by TLR ligands are able to induce class-switch recombination in B cells, promoting the generation of plasma cells producing IgA, which are a subclass of immunoglobulins recognized to induce a tolerizing phenotype at mucosal surfaces [34]. Collectively, the available data point to a role of lung microbiota in the creation of an immune-tolerant environment.

### Evidences of the essential role of lung microbiota in pulmonary immune tolerance

Among the human body non-sterile tracts, the presence of commensal bacteria is essential to maintain homeostasis and, in general, a good health status. To avoid chronic inflammation under normal condition, microbiota participate in shaping an immune-tolerant environment especially through the development, activation and recruitment of immune cells with immune-regulatory activity such as *T*_reg_, M2-polarized macrophages and tolerogenic DCs. Although this important role played by commensal bacteria is widely described in the context of intestinal immunity, today there are also some insights regarding lung immune contexture [35].

Indeed, lung microbiota have been recently thought to play a role in the immune tolerance influencing the recruitment and activation of APCs and *T*_regs_. This hypothesis is supported by multiple lines of evidence both in mice and in humans. In mice, Gollwitzer et al. have demonstrated that the appearance of specific bacterial taxa after birth is necessary for the development of *T*_regs_ [36]. Immediately after birth, newborn mice were susceptible to develop excessive airway eosinophilia, accompanied by the release of T-helper two (Th_2_) cytokines and airway hyper-responsiveness after exposure to house dust mite allergens, although their lungs had great quantities of CD4^+^Foxp3^+^CD25^+^Helios^+^*T*_reg_ cells. During the first 2 weeks after birth, the bacterial load in the lungs increases, paralleled by a progressive shift of bacterial phyla from a prevalence of *Gammaproteobacteria* and *Firmicutes* toward *Bacteroidetes.* The modifications of microbiota composition determine a decreased responsiveness to aeroallergen due to the appearance of Helios–T_reg_ cell subset that exerts potent immunosuppressive activity. The development of this population depends on the increased expression of programmed death-ligand-1 (PD-L1) on DCs, induced by the changes of lung commensal community. Lack of microbial colonization or PD-L1 blockade during the first 2 weeks after birth caused an excessive sensitivity to allergens that continued until adulthood. Adoptive transfer of *T*_reg_ cells from adult mice to newborn animals, before aeroallergen exposure, reversed hypersensitivity [36]. Moreover, it has been reported that germ-free (GF) mice sensitized and challenged with ovalbumin have an increased airway hyper-reactivity and inflammation compared to specific pathogen-free (SPF) mice and that the increased reactivity is reduced after reconstitution with commensal bacteria [37]. This study revealed that the immune dysregulation in GF mice was associated with alterations in plasmacytoid DCs (pDCs) and AMs development and function in the absence of commensals. Therefore, the commensal flora “educates” lung immune cells, shaping them toward a Th_2_-prone activation state. The observation that exaggerated allergic airway inflammation can be reverted by the reconstitution of GF mice with lung commensals obtained from wild-type mice may represent the rationale for the development of new possible therapeutic strategies in the treatment of allergic airway inflammation [37].

Moreover, Wang et al. demonstrated that SPF mice are more sensitive to death induced by acute inflammation after influenza virus challenge than mice living in a natural environment. The authors demonstrated that the presence of commensal *Staphylococcus aureus*, commonly colonizing the upper respiratory tract (URT), is essential for resistance to lethal inflammatory response [38]. The mechanism underlying the protective effect mediated by *S. aureus* relies in the recruitment of CCR2^+^CD11b^+^ monocytes from the bloodstream into the alveoli and the subsequent maturation and polarization to M2 AMs in a TLR2 pathway-dependent manner. Indeed, TLR2 deficiency or AM depletion abrogates this protection. In turn, M2 AMs suppress influenza-mediated lethal inflammation through the release of anti-inflammatory molecules and the expression of immunomodulatory ligands [38]. This work opened a new and intriguing scenario in which the airway microbiota acts as defender against influenza-mediated lethal inflammation.

As described, most of the data presented relies on the use of GF mice. This preclinical model has the undoubted advantage of allowing the dissection of the immunological effect exerted by one or more bacteria. However, it should be noted that these mice carry intrinsic biases that might represent confounding factors during the interpretation of the results. Although it has been demonstrated that GF mice have a similar level of B and T cells, conventional and CD103^+^ DCs and pDCs compared to normal mice [36, 39], the lack of microbiota interferes with the correct development of the immune system [36, 37]. Therefore, in these mice the immune system is quite immature, and the introduction of bacteria may induce an immune response that it is not superimposable with a “physiologic” inflammatory response [40, 41]. Alterations in the immune response in these mice are also testified by the fact that they do not respond to different types of immunotherapy [42, 43].

The ability of lung microbiota to mitigate the inflammatory response was also observed in humans. Indeed, AMs isolated from healthy subjects, divided into two different groups (pneumotypes) according to lung microbiota composition, responded differently to TLR4 ligation. Particularly, the presence of high bacterial load and predominant taxa derived from URT was associated with an attenuated immune response of AMs to lipopolysaccharide (LPS). This result indicated a role of microbiota in regulating the inflammatory response at the pulmonary mucosal surface [44]. Other studies in humans indicate that the presence of members of the *Bacteroidetes* phylum decreases lung inflammation [45], while *Prevotella *spp*.* and *Veillonella *spp*.* increase T-helper 17 (Th_17_) cell-mediated lung inflammation [44]. Studying a cohort of patients affected by HIV and pneumonia, Shenoy et al. were able to classify patients into distinct groups based on similarities of airway microbiome composition and demonstrated that a specific microbiome was associated with a down-regulation of T-helper 1 (Th_1_) proinflammatory responses and chronic viral infection [46]. All these studies indicate that commensal microorganisms that colonize the respiratory tract are critical in maintaining and sustaining immune “tolerance” in the lung microenvironment.

## Lung microbiota and cancer

The available data, obtained comparing the lung microbiota of healthy subjects versus patients with different lung pathologies, revealed significant differences in lung commensal composition, even in lung cancer patients. As supported by different studies, the alpha diversity (the number {richness} and distribution {evenness} of taxa expected within a sample) is generally significantly higher in healthy than in tumor lung tissues even if several taxa have been shown to be enriched in cancer specimens. For instance, by analyzing 210 bronchoscopic samples, Laroumagne et al. identified Gram-negative bacteria such as *Haemophilus influenzae, Enterobacter *spp*.* and *Escherichia coli*, colonizing lung cancer [47], while Hosgood et al. found an enrichment of *Granulicatella, Abiotrophia* and *Streptococcus* genera in oral and sputum samples from lung cancer patients compared to healthy controls [48]. Recently, in a study on salivary samples from 20 lung cancer patients and 10 control subjects, the genera *Veillonella, Neisseria, Capnocytophaga* and *Selenomonas* were reported to be significantly altered in patients with squamous cell carcinoma and adenocarcinoma [49]. This work also showed that the evaluation of the presence of both *Veillonella* and *Capnocytophaga* might be useful as a tool for lung cancer diagnosis [49]. In another study, *Firmicutes* and *TM7 phyla* and *Veilonella and Megasphera* genera were more abundant in bronchoalveolar lavage (BAL) of patients with lung cancer as compared to patients with benign diseases [50]. These results point again to the utility of the analysis of specific bacteria phyla or genera as potential biomarkers for the diagnosis of lung cancer. Furthermore, *Streptococcus viridians* together with 16 other microbial species were higher in lung cancer patients, while 7 species were specific of healthy controls [51].

Differences in terms of microbiota composition may be not restricted to the dicotomy “cancerous versus healthy” tissues, but there are also clues that the presence of a growing tumor can affect the microbiota also in parts of lung parenchyma not invaded by neoplastic cells. For instance, Liu et al. compared the lung microbiota of paired samples from tumor tissues and the contralateral noncancerous sites collected from 24 lung cancer patients and samples from 18 healthy subjects [52]. A significant decrease in microbial diversity was observed in patients with lung cancer compared to controls. The genera *Streptococcus* and *Staphylococcus* were found significantly more abundant in cancer and control subjects, respectively. Interestingly, *Staphylococcus* and *Dialister* abundance peaked in healthy samples, gradually declined in noncancerous tissue of lung cancer patients and reached the lowest presence in tumors. Collectively, these data indicate that microbiota profile profoundly changes during carcinogenesis and that tumor cells create for themselves a favorable microenvironment also impacting on the global lung microbiota by a mechanism not completely understood [52]. However, a recently published paper by Segal’s group provided some insights about the mechanisms through which microbiota can favor cancer cell development and growth. They showed that lung cancer patients were characterized by an enrichment of the taxa *Streptococcus* and *Veillonella* in their lower respiratory tract compared to the control group. Parallel transcriptomic analysis of bronchial epithelial cells revealed an up-regulation of the ERK/PI3K pathway and in vitro experiments demonstrated that *Veillonella* was able to directly promote the activation of ERK/PI3K signaling in airway epithelial cells and in lung tumor cells. This pathway is known to represent an early event in lung carcinogenesis and, therefore, these findings may provide one the first mechanistic explanations about how a dysregulated microbiota can influence tumorigenesis [53]. The main findings of the presented clinical studies are summarized in Table 1.

**Table 1:** Microorganisms found in lung cancer patients: clinical studies

Authors	Reference	Type of sample	No. of subjects	Findings
Laroumagne S. et al	47	Lung aspirates by bronchoscopy	210	*Haemophilus influenzae*, *Enterobacter* spp. and *Escherichia coli*
Hosgood HD III et al	48	Buccal samples and sputum samples	16	*Granulicatella*, *Abiotrophia* and *Streptococcus*
Yan X. et al	49	Saliva samples	86	*Capnocytophaga*, *Selenomonas*, *Veillonella* and *Neisseria*
Lee SH. Et al	50	Bronchoalveolar fluid	28	Phyla: *Firmicutes and TM7*Genera: *Veillonella* and *Megasphaera*
Cameron SJS. Et al	51	Spontaneous sputum	10	*Streptococcus viridans* and 16 other microbial species
Liu HX et al	52	Bronchoscopy samples	42	*Streptococcus*

There are also some evidences of the potential relationship between lung microbiota composition and lung cancer prognosis. In particular, Peters et al. in a pilot study, performed on paired lung tumor and distal normal samples from the same lobe/segment of 19 patients with non-small cell lung cancer (NSCLC), showed that higher diversity and alteration in lung commensal bacterial composition of the unaffected lung tissue was associated with reduced disease-free (DFS) and recurrence-free (RFS) survival [54]. In the same work, greater abundance of *Koribacteraceae* was associated with increased RFS and DFS, whereas greater abundance of *Bacteroidaceae, Lachnospiraceae* and *Ruminococcaceae* was associated with reduced RFS and DFS) [54] (Table 2).

**Table 2:** Association between lung microbiota and lung cancer prognosis

Authors	Reference	Type of sample	No. of subjects	Findings
Peters HA et al	54	Lung cancer and matching normal lung tissues	19	*Koribacteraceae* associated with increased RFS and DFS
				*Bacteroidaceae*, *Lachnospiraceae*, and *Ruminococcaceae* associated with reduced RFS or DFS
Yu G et al	55	Lung tissue samples	165	Thermus more abundant in advanced stage (IIIB, IV) patients
				*Legionella* higher in patients developing metastases

*RFS* recurrence-free survival, *DFS* disease-free survival

These data point out that bacteria in resected normal lung tissue might serve as biomarkers to predict disease recurrence risk in early-stage NSCLC. If the prognostic value of lung microbiota composition is validated in future studies, it will also represent a novel target for therapeutic intervention to improve RFS in lung cancer patients (Table 2).

An association between the presence of specific bacteria and clinical outcome has been also reported in a cohort of 165 non-malignant lung tissue samples collected from lung cancer patients with different stage disease. The genus *Thermus* was more abundant in tissue from advanced stage patients (IIIB, IV), while *Legionella* was higher in patients who developed metastases [55]. Again, the non-malignant lung tissue was characterized by higher microbiota alpha diversity than the matched tumor tissue, confirming the results above reported.

The relationship between the lung microbiota and lung cancer has been investigated in different epidemiologic studies, and it has been suggested that microbiota alterations may also play an important role not only during tumor progression, but also in the response to therapy [56]. However, most of the published data on the impact of microbiota in regulating the efficacy of different types of drugs were obtained studying bacteria in the gut [57] and, to the best of our knowledge, there are no reports regarding lung microbiota. However, since many anticancer agents are systemically administered, they can easily reach the lung environment through the bloodstream and, here, can be uptaken by commensal bacteria and chemically modified. Although the metabolism of lung microbiota is far from being understood, few data suggest that microorganisms inhabiting the lung may possess metabolic activity themselves. For instance, in the previously cited paper by Segal et al., the authors found that people bearing a microbiota defined as Pneumotype_SPT_ were characterized by a peculiar metabolic profile in lungs [44]. Moreover, Cribbs et al. observed metabolomic differences in BAL obtained from HIV-infected patients compared to healthy controls and these differences were ascribed to the presence of *Caulobacteraceae*, *Staphylococcaceae* and *Nocardioidaceae* in HIV individuals [58, 59]. Therefore, bacteria in the lungs might be a metabolically active consortium able to modify the chemical structure of therapeutic agents altering their pharmacological activity (in a positive or negative way) and/or affecting their effective local concentration [60, 61].

Some indications suggest a causal role of specific bacteria in promoting cancer development. A significant relationship between *Mycobacterium tuberculosis* (TB) and development of lung cancer has been found [62]. A mechanistic explanation of this association may be related to a possible tuberculosis-induced chronic inflammation-associated carcinogenesis [62]. The effect of microbiota in inducing carcinogenesis probably begins with the ability of microbiota and its metabolites to activate TLRs in immune and epithelial cells resulting in the initiation of an inflammatory processes that, if persistent, can cause irreversible damages in normal cells and promote the occurrence of tumors [56].

Another important aspect to be considered is that bacteria within the tumor microenvironment influence cancer cell growth by exerting immunomodulatory effects.

We previously observed that the aerosol delivery of various agents, such as TLR agonists and cytokines, was able to favor the immune-mediated control of experimental lung metastases, indicating the feasibility of influencing local lung immunity through a non-invasive strategy [63-67].

Based on this evidence, we recently reported that the commensal lung microbiota can be in vivo manipulated by aerosolized antibiotics. This treatment, decreasing the percentage and activity of T_regs_ and, at the same time, activating immune effector cells, enhanced immunosurveillance in the lung and reduced the growth of B16 melanoma lung metastases [68]. T_regs_ reduction was associated with a decrease of abundance of the genus *Streptococcus* and the concomitant expansion of generally less represented genera of *Proteobacteria* and *Actinobacteria* [68]. Moreover, we also demonstrated that aerosolized *Lactobacillus rhamnosus* promote immunosurveillance against melanoma metastases by inducing the maturation of resident pulmonary APCs and reducing the M2 polarization of alveolar macrophages [68]. Thus, the two different strategies to modify the lung microbiota by aerosolizing antibiotics or probiotics, promote an anti-tumor activity with different immune mechanisms are: antibiotics activate antitumor effector cells by inducing a pauperization of microbial signals necessary for the recruitment of *T*_regs_ in the lung, while probiotics appear to overcome the commensal bacteria-induced tolerance promoting the maturation of resident APCs [68]. Moreover, besides playing an antitumor effect through the modulation of microbiota, some antibiotics, as macrolides, also exert pharmacological effects directly on tumor cells, particularly reducing pro-inflammatory cytokines release and inhibiting autophagy and angiogenesis [69]. In a study on NSCLC patients, indeed, clarythromicin treatment was associated with an increased survival time and improved benefit of patients, ascribed to reduced IL-6 levels [69]. Thus, this direct antitumor activity of macrolides, by influencing the tumor microenvironment, might in turn result in further shaping of the lung microbiota.

In agreement with our results, a recently published paper revealed that in a genetic model of lung cancer, antibiotic-treated or germ-free mice are protected from lung cancer development [70]. In that study, a low bacterial burden in the lungs (but not in the gut) significantly correlated with reduced tumor growth, revealing a clear association between local microbiota abundance and lung cancer development. The augmented bacterial load and reduced diversity of tumor-associated microbiota was accompanied by the recruitment of IL-17-producing γδ T cells which, in turn, promoted pro-tumoral neutrophil expansion and tumor cell proliferation [70]. It should be also noted that the delayed tumor growth observed in GF mice may be also ascribed to the complete absence of tolerogenic mechanisms in lungs [36, 37], such as the presence of cells with immunoregulatory activity that could be exploited by cancerous cells to avoid immunosurveillance. They also demonstrated that intratracheal instillation of a consortium of bacteria isolated from late-stage lung tumors accelerated tumor growth and that local administration of bacterial products (LPS and peptidoglycan, PGN) robustly stimulated γδ T cells in the lung. Similar findings were also obtained in other tumor models where, again, a particular microbiota composition was associated with the presence in the tumor microenvironment of immunosuppressive and pro-tumorigenic immune cells [71].

Collectively, these results represent a strong scientific rationale to design new therapeutic strategies able to re-establish an antitumor immune response to modify the intratumoral microbiota.

It is worth pointing out that, as summarized in this section, the majority of the presented data concern the relationship between lung microbiota and lung cancer development and progression. Although few reports suggest that lung microbiota can affect the efficacy of different anticancer drugs, as in the intestine, this field is still at its infancy and needs to be further explored.

### Lung microbiota, non-oncology lung diseases and cancer

As described for lung cancer, also non-oncologic lung pathologies (i.e., chronic obstructive pulmonary disease, cystic fibrosis, lung infections, emphysema) can have an impact on lung microbiota composition that, although different for each situation, is characterized by loss of bacteria abundance and diversity paralleled by an increased presence of certain phyla such as *Proteobacteria* (mostly *Neisseria*, *Moraxella* and *Haemophilus*) [72]. For example, chronic obstructive pulmonary disease (COPD) is a chronic inflammatory disease that causes obstruction of the airways, hypersecretion of mucus and a variety of other symptoms leading to an impairment of lung parenchyma function [73]. Hilty et al. analyzed 43 bronchial lavages obtained from healthy individuals and patients with COPD or asthma by 16S rRNA sequencing and they observed that *Proteobacteria*, in particular *Haemophilus* spp., were more abundant in asthmatics or COPD patients than in healthy individuals. On the contrary, Bacteroidetes, mostly *Prevotella* spp., were more frequent in the control group [3]. Moreover, another study performed on patients with COPD, smokers with no airway disease and healthy subjects revealed the prevalence of members of *Pseudomonas* spp. in COPD patients, while *Prevotella* spp. was, again, more present in the healthy group [74]. This scenario is also complicated by the fact that lung microbiome differs with the severity of the disease [75-77]. A similar situation has been also described for cystic fibrosis [72]. Since many lung disorders represent a risk factor for the development of lung cancer [78] and all of these diseases shared the characteristic of an altered lung microbiota [72], it is highly plausible that these changes may represent one of the possible causes of tumorigenesis. This is particularly clear in the context of *Mycobacterium tuberculosis* infection, which determines chronic inflammation and lung tissue fibrosis correlated with lung cancer onset [62]. Chronic inflammation in the lungs can be also caused by other bacteria such as *Haemophilus*, *Moraxella*, *Streptococcus*, *Staphylococcus* and *Pseudomonas* [79]. On the other hand, the pattern of bacteria characterizing lung cancer patients and subjects with other lung diseases is different [80], suggesting that lung dysbiosis can induce chronic inflammatory status that, in turn, may trigger and sustain cancerogenesis. Subsequently, the presence of a growing tumor may further shape lung microbiota composition.

### Lung microbiota, smoke and cancer

Cigarette smoke is one of the main causes of morbidity and mortality all over the world and it is associated with different types of lung disease, especially lung cancer [81]. Smoking has a profound impact on upper airway microbiota, but it appears that it cannot have the same effect on the lower respiratory tract bacteria composition. Indeed, Morris et al. analyzed 64 bronchoscopic alveolar lavages collected from non-smokers and smokers and they found differences between the two cohorts only in mouth bacteria communities but not in lungs [82]. Moreover, Munck et al. reported the lack of important changes in bacterial diversity after smoking cessation [83], indicating that smoking does not have a key role in determining modifications in lung commensal composition. On the other hand, studies performed in humans [74] and mice [84] suggested that smoke exposure can alter bacterial composition in the lower respiratory tract, probably by impairing local immune cell activity. Since chemical compounds present in the tobacco smoke may affect lung immune cells function [85, 86], smoking may determine a disequilibrium in the microbiota–immune cell cross talk, leading to dysbiosis, chronic infection and, eventually, lung cancer.

## Implications and perspectives

Although the resident lung microbiota has been described only recently, its association with lung cancer development is clearly emerging. The data regarding a link between lung microbiota and lung cancer development open a key question that remains to be answered: “can lung microbiota be the cause or the consequence of lung cancer development?” Based on the available data, it is not possible to draw any final conclusion but some clues allow us, at least, to speculate. First, the colonization of lungs with particular microbial species can be causally related to chronic inflammation and lung carcinogenesis. In normal conditions, it seems that lung microbiota is involved in promoting immunesuppressive cell accumulation to prevent unwanted inflammatory reactions to harmless stimuli. On the other hand, patients with pulmonary diseases, reported to be risk factors for lung cancer, have a microbiota that profoundly differs from that of healthy lungs that, indeed, determines a chronic inflammatory status. In this scenario, particular strains of bacteria may alter the physiologic immunotolerant environment by “reprogramming” resident immune cells, for example through the release of particular metabolites with immunoregulatory properties, or by recruiting inflammatory immune cells from the bloodstream. Second, it is also true that tumor cells can modify lung environment and these alterations may, in turn, impact on lung microbiota composition. Future studies will answer this question and reveal whether the lung microbiome can be manipulated therapeutically to influence disease progression and the response to therapy.
